# What a Plant Sounds Like: The Statistics of Vegetation Echoes as Received by Echolocating Bats

**DOI:** 10.1371/journal.pcbi.1000429

**Published:** 2009-07-03

**Authors:** Yossi Yovel, Peter Stilz, Matthias O. Franz, Arjan Boonman, Hans-Ulrich Schnitzler

**Affiliations:** 1 Animal Physiology Department, University of Tuebingen, Tuebingen, Germany; 2 University of Applied Sciences, Konstanz, Germany; 3 INCM-CNRS UMR, Marseille, France; University College London, United Kingdom

## Abstract

A critical step on the way to understanding a sensory system is the analysis of the input it receives. In this work we examine the statistics of natural complex echoes, focusing on vegetation echoes. Vegetation echoes constitute a major part of the sensory world of more than 800 species of echolocating bats and play an important role in several of their daily tasks. Our statistical analysis is based on a large collection of plant echoes acquired by a biomimetic sonar system. We explore the relation between the physical world (the structure of the plant) and the characteristics of its echo. Finally, we complete the story by analyzing the effect of the sensory processing of both the echolocation and the auditory systems on the echoes and interpret them in the light of information maximization. The echoes of all different plant species we examined share a surprisingly robust pattern that was also reproduced by a simple Poisson model of the spatial reflector arrangement. The fine differences observed between the echoes of different plant species can be explained by the spatial characteristics of the plants. The bat's emitted signal enhances the most informative spatial frequency range where the species-specific information is large. The auditory system filtering affects the echoes in a similar way, thus enhancing the most informative spatial frequency range even more. These findings suggest how the bat's sensory system could have evolved to deal with complex natural echoes.

## Author Summary

More than 800 species of bats perceive their surroundings through echolocation. They emit ultrasonic pulses and analyze the information conveyed in the echoes returning from objects in their surroundings. This enables bats to orient in space, to acquire food and to perfectly function in complete darkness. In the absence of light, echoes constitute a major part of the sensory world of bats. Understanding their characteristics can thus help to shed light on the echolocation sensory system. The goal of this work is to study the characteristics of natural plant echoes. Plant echoes are very abundant in the world of the bats and are used by them to find food sources and to navigate. We show that some of the features of the echoes can be explained from the physical properties of the plant they were sampled from (e.g., its leaf size and density). We then analyze the effects of the sensory system on the echoes and suggest that they improve the representation of the echoes in a way that enhances the information that is most reliable for the bats.

## Introduction

The understanding of natural stimuli is critical for understanding the sensory systems that evolved to process them. More than 800 species of echolocating bats continuously emit echolocation signals and analyze the returning echoes to perceive their surroundings [1],[2]. Without light, natural echoes form a major part of the sensory world of these bats. As such they may have played an important role in the design of echolocation signals during evolution as well as in the design of the vocal apparatus, the receiving mechanisms and the computational processes in the bat's brain. Despite their significant importance, the characteristics of natural echoes are very poorly understood. In the field of vision, substantial efforts have been invested in characterizing natural images [3],[4]. These efforts led to a better understanding of the sensory system (e.g. the retinal ganglion cells or large monopolar cells in the fly [3]–[8]). In this study we investigate the acoustical sensory world of bats. We aim to set a framework to analyze the statistics of natural echoes as available for echolocating bats.

Although vegetation echoes, on which we focus in this work, are among the most common echoes bats constantly encounter, they have been scarcely studied [9],[10]. Bats interact with vegetation in many different ways. In some cases such as feeding from a flower, landing on a fruit or avoiding collision with a branch, the bat must accurately localize the position of the relevant part of the plant. In other cases bats might use plant echoes that were acquired from a distance of a few meters. Such situations include two of the bat's most fundamental tasks: spatial orientation and food acquisition. In order to navigate from their roost to the foraging sites and vice versa, bats must use landmarks. Plants present one of the most common acoustical landmarks and echoes from their edges might facilitate the route following behavior in bats [11]. Additionally, certain types of vegetation such as meadows, bushes, trees etc. may be indicators of specific sources of bat food [12],[13]. Some evidence exists that bats respond to vegetation echoes that are acquired from a distance of several meters and are able to use them. H-U Schnitzler and A Denziger [unpublished data] trained Natterer's bats to discriminate conifers from broad-leaved trees and observed that horseshoe bats commuting along a hedge of bushes show distinct reactions in their echolocation behavior when the reflection properties of the bushes are changed by covering them with velvet.

Since plants have complex shapes that cannot be described in terms of simple geometrical primitives vegetation echoes exhibit highly complex temporal patterns [14]. From an acoustical point of view, a plant can be approximated as a stochastic array of reflectors formed by its branches and leaves. In all the behavioral tasks mentioned above, bats ensonify the vegetation from a relatively long distance of up to a few meters. At such a distance the width of the sonar beam impinging on the vegetation is so large that the echo returning from the plant is a stochastic superposition of numerous reflections from reflectors in a large variability of sizes, distances and orientations. This variability makes it futile to rely on characteristic spectral notches created by interference of overlapping echoes, as is commonly done in the case of simpler objects [15]. There is also no point in describing the echo in terms of the positions of single reflectors. This can change largely depending on the specific specimen and ensonification angle. In addition, the large differences between the long audio streams received by the two ears makes it extremely difficult in most cases to identify the two corresponding glints of a single reflector and therefore very hard to localize it. Thus, the observed ability of bats to use the information contained in these echoes (see above and [16]) must rely on statistical features. In the following, we will study some of the statistical properties of vegetation echoes which might play a role in bat behavior. We will examine both spectral and temporal statistics using the power spectra of the echoes and the power spectra of the envelope of the impulse responses. We will try to connect their statistics to the physical properties of the plants they were sampled from. We will investigate the effect of the echolocation system and the early auditory processing on the statistics of these echoes. Finally, we will show that these latter spectra can be described by a simple Poisson model of the spatial reflector arrangement.

### Spectral statistics

The filtering characteristics of a reflecting object are known as its frequency response and mainly depend on its acoustical cross-section. In a plant covered with leaves, the leaves will reflect most of the energy emitted by the bats [9]. The cross section of the leaves will have a major influence on the spectra of the echoes. Basically (see [17] and [18] for more details), when the circumference of a spherical reflector is much larger than all of the wavelengths of the emitted signal, the intensity of the reflection is equal in all frequencies (optic domain). If the circumference of the reflector is smaller than the wavelength, however, the intensity of the reflections decreases rapidly when decreasing the radius of the sphere (Rayleigh domain). A sphere between these two domains with a circumference of the same order as the wavelength will reflect energy according to the ratio between its circumference and the wavelength (resonance domain). Leaves are not spheres, but the same reasoning applies to them. Broad-leaved plants will have less spectral filtering effects on the echoes than small leaves or needle-like leaves that act as high pass filters. The analysis of the spectral statistics is therefore straightforward. Since the spectrum of the emitted signal is always identical in all experiments, differences between the spectra of different species imply structural differences and particularly differences in the reflectors cross section.

### Temporal statistics

The spatial arrangement of the reflectors relative to the receiver determines the temporal statistics of the echoes. The spatial arrangement of the branches also plays an important role since it defines the arrangement of the leaves. The analysis of the temporal statistics of the echoes is not as straightforward as that of the spectral statistics. There are many possible parameters that represent the temporal statistics. We chose to use parameters that are strongly related to the spatial distribution of the reflectors, i.e., to the structure of the plant. Additionally, in order to be comparable to the findings in natural images we use a similar method to the one used to characterize them [3],[4].

**Figure 1:**
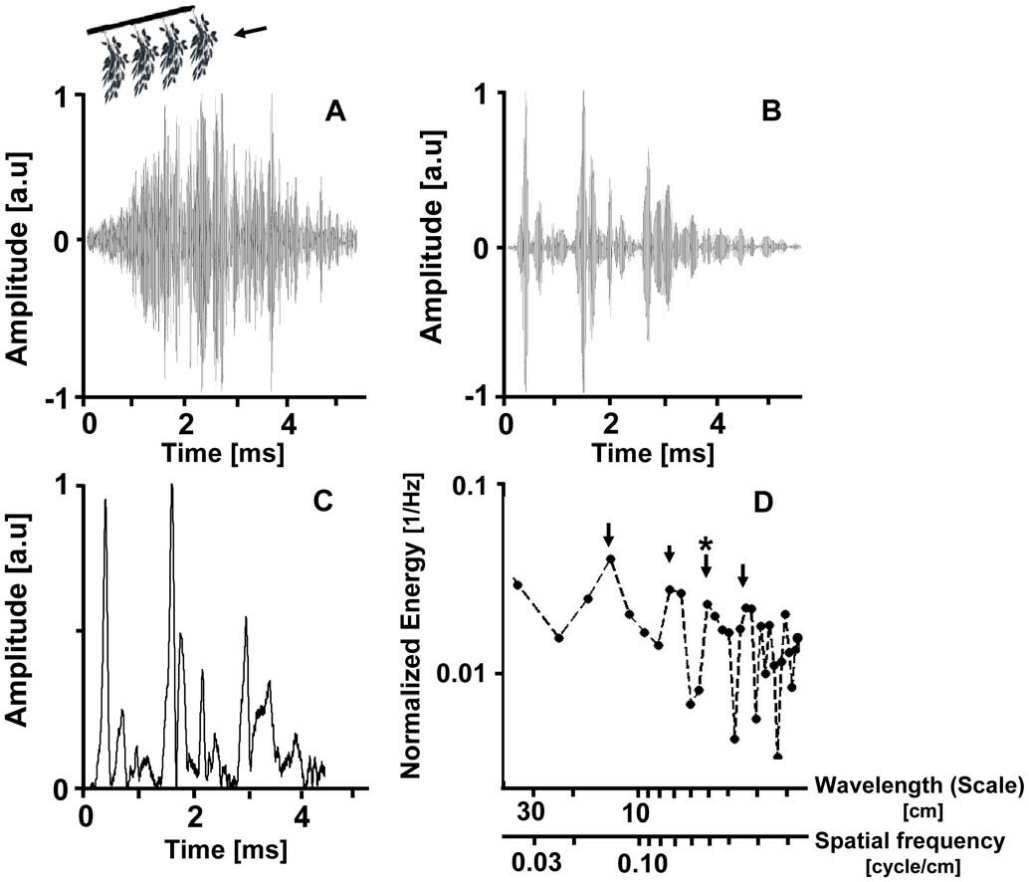
The basic assumption. A) The normalized echo train returning when ensonifying an artificial branch with four identical plastic leaved twigs 15 cm apart from a flat horizontal angle (see illustration above A. a.u – arbitrary units. B) The normalized IR of the echo train presented in A. C) The normalized envelope of B. Note the clear correspondence to the structure of the plant. The envelope presents three main peaks ∼0.9 ms apart corresponding to the first three twigs. The echo from the fourth twig is much weaker due to occlusion. Additional smaller peaks follow each of the main ones and correspond to the leaves on the twigs. D) The PSD of C shows a main peak around 14 cm, as expected, with following peaks at the higher harmonics at, e.g., 7, 3.5 and 1.8 cm. One peak however, at ∼5 cm (marked by an asterisk) deviates from this sequence and is probably a result of the periodic structure created by the leaves on the twigs. The energy (Y-axis) is normalized to a sum of one.

We base our analysis on the envelope of the impulse response [IR, see Figure 1B and 1C] of the echo. The IR is defined as the output of a system (i.e., the plant) when presented with a Dirac delta function. The envelope of the IR is directly related to the spatial arrangement of the reflectors and can be intuitively thought of as a one-dimensional image of the plant. Peaks in the envelope correspond to glints returning from reflectors. The time between them equals twice the time that the signal travels between these reflectors. The distance between them relative to the receiver is therefore the distance corresponding to half of this time (according to the speed of sound). We used the Power Spectral Density (PSD, Figure 1D) of the envelope to characterize the abundance of reflectors (glints) at different distances from each other. A PSD quantifies the amount of periodic structure in a signal over the entire frequency range. Due to the nature of the Fourier transform, the PSD of even a single glint will contain energies spread on the entire frequency range. However a sequence of repeating glints with a certain time difference between them constitutes a periodic structure resulting in a higher energy at the corresponding frequency in the PSD. Each frequency in the PSD corresponds to a cycle time that can be translated into a wavelength or a spatial distance according to the speed of sound:
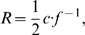
(1)where R is the distance between two reflectors; c is the speed of sound and 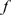 is the frequency. We will refer to this as the wavelength or the scale. The corresponding spatial frequency equals 1 over the wavelength.

The basic assumption of our analysis is that the energy of a certain frequency in the PSD correlates with the amount of reflectors in periodic structures with corresponding spatial distances between them (Figure 1D). Plants tend to have characteristic distances that correspond to periodic structures: Series of leaves, twigs, branches, etc create periodic structure with typical distances between them. Obviously the distance between two reflectors in comparison to the receiver depends on the angular relation of the three. Therefore, when ensonifying a periodic structure from a limited sector of angles the average PSD will not present a sharp peak at the corresponding distance, but rather a broad one around it.

When averaging the spectra of a plant from many different orientations, particular distances that are very common in the plant such as distances between twigs or larger branches will create broad peaks of energy around the corresponding frequency in the spectra. These distances are common from any direction the plant is observed. Uncommon distances, however, will affect the energies in all frequencies and will be negligible in relation to the common ones. If no such common distances exist we should receive a flat spectrum. Naturally there could be periodic structures that are very salient from a single orientation, but these clearly do not constitute a reliable feature for characterizing a plant species.

Throughout the paper we shall refer to spatial frequencies according to the wavelength of the corresponding periodic structure of reflectors, which we will refer to as the *scale* of this structure.

### Modeling the creation of complex echoes statistics

In an attempt to understand the vegetation properties that determine the temporal statistics of the echoes, we developed a computer model of the physical processes that underlie their creation. The model first produces a three-dimensional distribution of reflectors. Next, an echo is created as a superposition of the echoes returning from all of the reflectors with delays corresponding to their distance from the receiver (R), and with energies attenuated according to the geometric attenuation of point reflectors (1/R^4^). Modeling reflectors with a different cross-section would lead to the same temporal statistics but to a different frequency response.

Assuming that the spatial arrangement of the reflectors has the most important effect on the temporal statistics, we started with a simple model that only considers the reflectors distribution and does not take into account other possible factors such as leaf shape, orientation, occlusion etc.

The uniform Poisson model is the simplest reflector distribution we examined. It assumes that the reflectors are evenly distributed everywhere in the plant according to a Poisson distribution. Thus, if 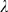 is the expected number of reflectors in a cubic volume with a side length of 0.5 cm (the basic unit of volume we used), then the probability of finding a single reflector within this volume is given according to the Poisson distribution:
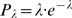
(2)We also examined more complicated models with reflectors distributed in clusters (non- stationary distributions). In our so-called second-order model, the centers of the clusters are first determined according to a Poisson distribution as described above. Next, Gaussian clouds of reflectors are created around these centers, with a probability of finding a reflector at a radius x from the center given by the Gaussian probability density:
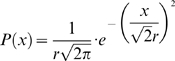
(3)r represents the radius of the cluster.

Higher-order models can be created in a sequential fashion by using the reflectors of the previous order as centers for smaller Gaussian clouds. These models can be intuitively thought of as a representation of the arrangement of reflectors in a plant, starting from the gross skeleton, bifurcating several times and finally ending at the leaves.

## Results

### Spectral statistics

**Figure 2:**
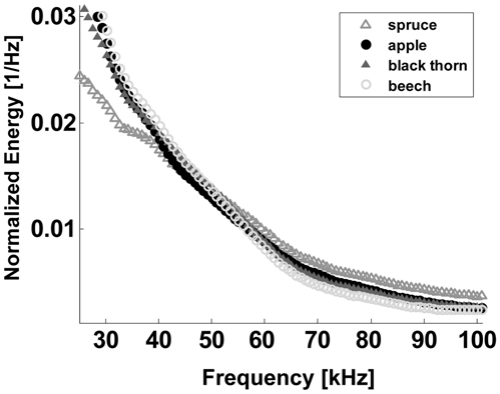
Mean normalized spectra of the echoes. The spectra were first divided by the emitted signal spectrum to calculate the relative reflected energy at each frequency. The result was then normalized to have a sum of one.

The average normalized spectra of the echoes for each of the investigated four species is shown in Figure 2. The differences between the species can be best understood in terms of the cross section of their main reflectors – the leaves. Spruce trees show the largest differences compared to the other plants as expected from their needle-like leaves. Their spectra contain the least energy in the low frequencies and the highest energy in the high frequencies due to their small reflectors. The three broad-leaved species spectra are similar. In the very low frequencies (<40 kHz) blackthorn has the lowest relative energy due to its smaller reflectors (see methods) while beech has the highest. In the intermediate range (>40 & <60 kHz) blackthorn shows the highest energy probably because most of its reflectors are in the optic region for this range. In the high frequencies (>60 kHz) beech exhibits the lowest energy while the other two are similar.

### Temporal statistics

#### The relation between the physical plant and its echoes

**Figure 3:**
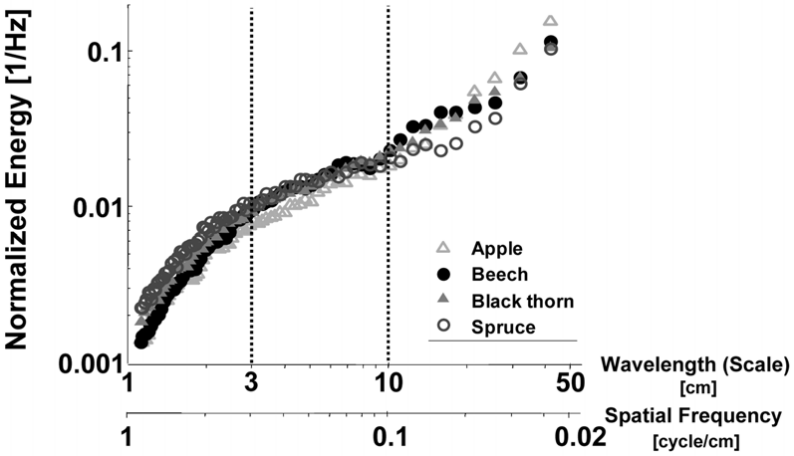
Mean normalized spectra of the IR envelopes of four plant species common in the central European bat environment. Spectra were normalized to have a sum of one.

**Figure 4:**
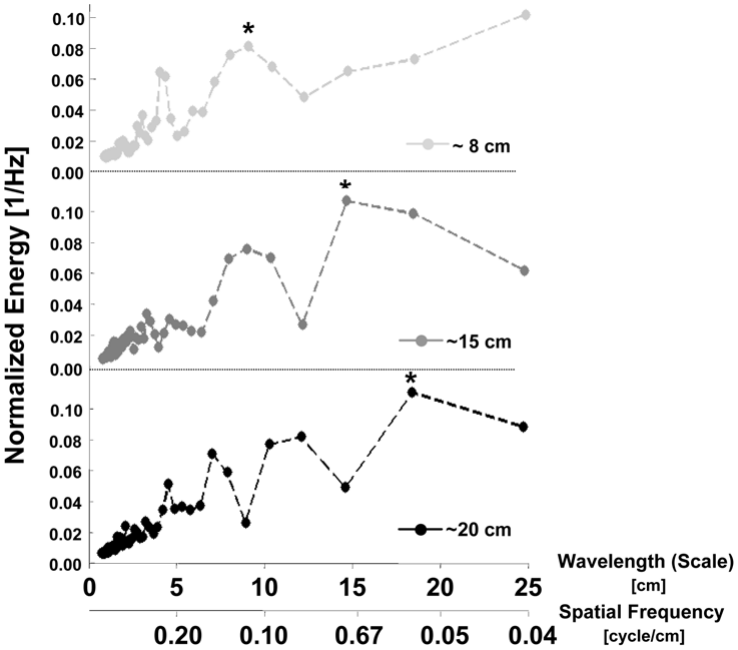
Mean normalized spectra of the envelope of the echo train returning from an artificial branch. Each branch was composed of a row of 4 artificial plastic twigs which were 8, or 15 or 20 cm apart. Notice the energy peaks around 8, 15 and 20 cm in the three graphs (marked with asterisks). Spectra were normalized to have a sum of one. We present the spectra on a linear scale here in order to emphasize the peaks of energy corresponding to the distance between twigs.

**Figure 5:**
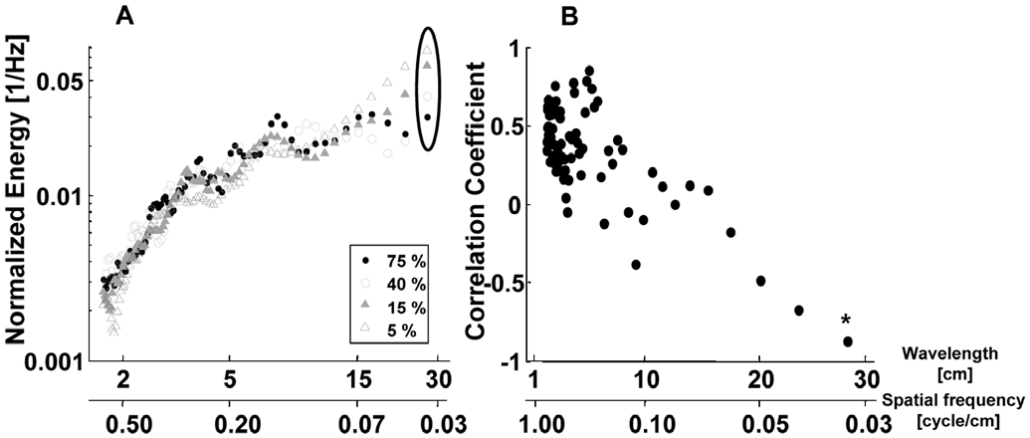
The effect of the leaf density on the echoes' spectra. A) Mean normalized spectra of the envelopes of the echo trains of a *Ficus* plant with four different leaf densities (out of the eleven measured). Notice the shift of energy from the small scales to the large scales as leaf density decreases. Spectra were normalized to have a sum of one. B) Pearson correlation coefficient between leaf density and energy. Each point in the graph presents the correlation between 11 leaf densities and their corresponding energies for the scale given on the X axis. As an example, we marked the correlation between density and energy at a scale of 27 cm (depicted by an ellipse in A and an asterisk in B) that is almost perfectly negative linear.

* Natural plants: In the second analysis we examine the normalized spectra of the envelopes of the IRs. When presenting the averaged spectra on a doubly logarithmic plot, a surprisingly robust pattern emerges: all of the plant species we examined have an overall inverted sigmoid shape consisting of three approximately constant-slope domains in a spatial scale range corresponding to 1.7–50 cm, (Figure 3). The borders between the domains can be grossly placed around 10 cm and 3 cm. In spite of their similar overall shape, the spectra differ in their exact borders, their typical slopes and their absolute values. The most salient differences appear in first domain (>10 cm, this is seen better on a linear plot). We quantified this by calculating the average difference after normalizing according to the standard deviation. As discussed in the introduction and as will be demonstrated below, we hypothesize that these differences are a result of species-specific structure characteristics at different scales, particularly related to the frequent occurrence of certain distances between reflectors. In the two following experiments, we directly examine this hypothesis.

* Plastic model: To verify our basic assumption that a peak in the spectrum of the IR envelope corresponds to a common inter-reflector distance, we examined the echoes from a plastic plant model consisting of four broad-leaved twigs in a row perpendicular to the impinging sound. The peak of energy in the mean spectrum of the plastic branch echo envelope over a variety of ensonification angles indeed corresponds to the distance between the four twigs (Figure 4). This confirms our basic interpretation of the IR as a one-dimensional representation of the spatial arrangement of the reflectors, at least for such a simple structure when ensonified from a limited range of angles.

* Leaf density: To confirm that the differences between the sigmoid curves can be related to the complex spatial structure of the species, we examined the influence of the leaf density on the spectra of a *Ficus* plant. These spectra behaved differently in the different scale domains when decreasing leaf density (Figure 5). In the large scale domain (>10 cm) energy was negatively correlated with leaf density, i.e., there was an increase in the normalized spectral energy when the leaf density was lower. In the other two domains (<10 cm) however, there was a positive correlation meaning that the spectral energy decreased when lowering the leaf density (Figure 5B). Decreasing leaf density thus induces a shift of energy from frequencies representing small scale structures to those representing large scale structures. This confirms our hypothesis: continuously ripping off leaves in a random fashion on the one hand decreases the amount of nearby leaves and therefore the relative energy at small scales. On the other hand it increases the amount of leaves that are far from each other, and therefore the relative energy at the large scales.

McKerrow et al. [9] argued that leaves are the main reflectors in a plant while branches play only a minor role. Our experiments show that when decreasing the leaf coverage to nearly zero, the absolute energy of the echo indeed drops noticeably. In this sense our results confirm McKerrow's findings. However, although the leaves dominate the absolute energy of the echoes, the leaf arrangement is modulated by the branches. In this indirect way, branches exert a strong influence on the large scale periodic structures and their corresponding echo statistics. We therefore conclude that the leaf coverage amplifies the modulations in reflector density caused by twigs and branches.

#### The effect of the emitter on the statistics of plant echoes

**Figure 6:**
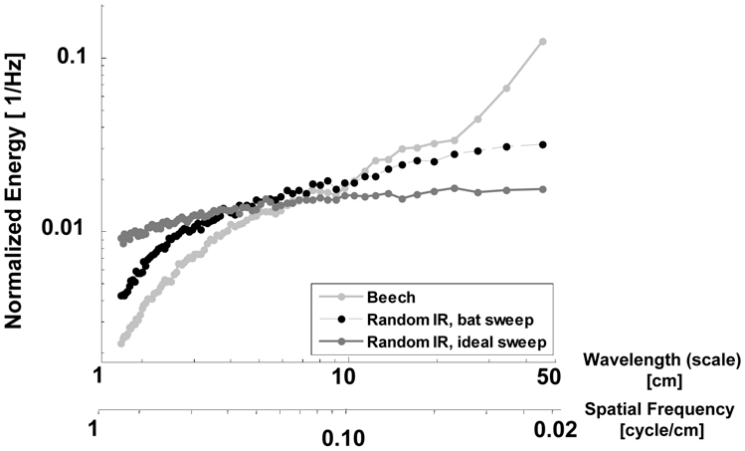
The effect of the emitter. Normalized spectra of the artificial IR-envelopes simulated with two different emitted signals aside the real spectrum of beech. Spectra were normalized to have a sum of one.

To study the effect of the emitted signal we generated artificial random IRs with a uniform distribution of distances (in the range 0–1 m) between the glints. Such an IR represents a plant that has an equal number of reflectors in all distances from any point of ensonification. These IRs were then convolved either with the emitted bat-like signal described above or with an artificial signal with a spectro-temporal structure that is identical to the emitted one, but with a constant energy in all frequencies (we call this the ideal bat sweep). We then ran the artificial echoes created by this process through the same analysis as the natural ones (Figure 6). If we used a Dirac delta function as the emitted signal, we would expect the spectra of the random IRs' envelopes to be flat since there is equal energy at all scales. This is almost the case for the ideal bat sweep with a slightly higher energy at the large scales. For the real emitted signal however, this bias is much more salient. The energy at large scales is clearly higher than at the small ones. The energy at the small scales (<6 cm) decreases logarithmically resulting in a shape that is very similar to the one that characterizes the real data.

#### The effect of the receiver on the statistics of plant echoes

**Figure 7:**
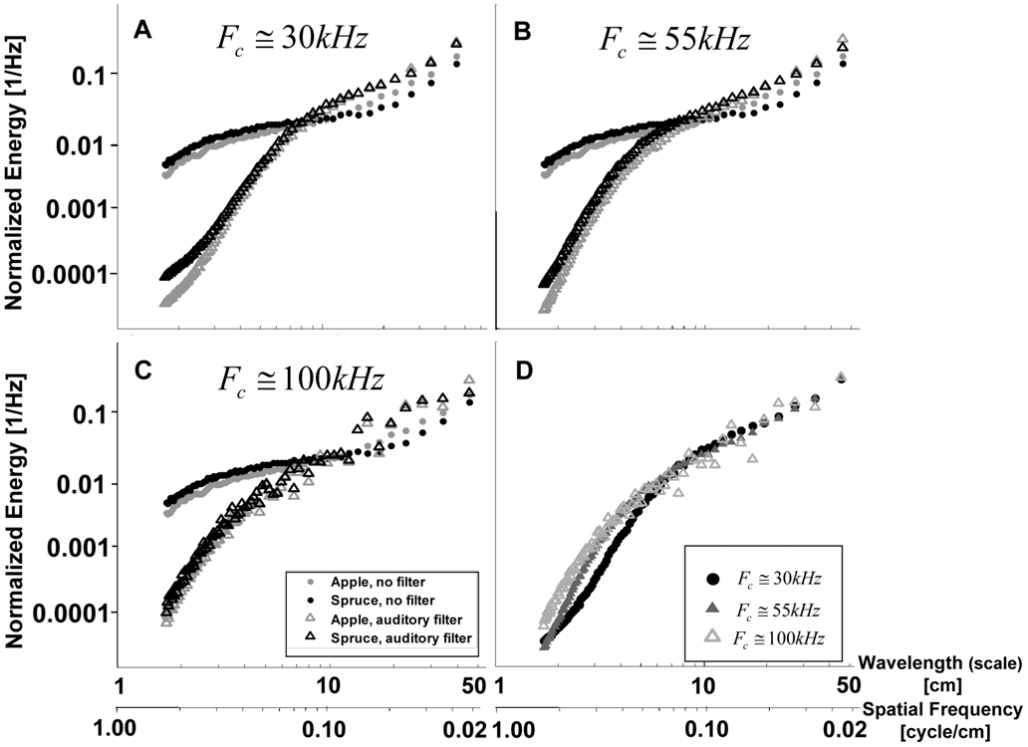
The effect of the auditory system. A–C) Normalized echo spectra of two species (apple and spruce) after auditory system filtering compared to the spectra of the same species with no filtering. The system response is represented by the output of three channels with different center frequencies (F_c_) representing the entire frequency range. (A) 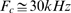, (B) 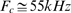, (C) 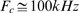. The energy was normalized to a sum of one. D) The output of the three channels of apple (appearing in A–C) are overlaid to emphasize the differences between them.

The temporal analysis presented so far was based on the envelope of the impulse response of the echoes. In order to have access to this information bats would have to be able to use a receiver that is equivalent to a non-coherent matched filter, yet such ability was never shown. In order to test the effect of the filtering of early stages in the auditory system on the statistics, we applied the commonly used auditory system model (see methods) on the raw echoes (Figure 7). We analyzed the spectra of the raw output signals of different channels of the auditory system representing the changes in the membrane potential of the outer hair cells of each channel. This information is actually not available to the animal directly. Instead it is encoded in parallel spike trains in the auditory nerve, which hold much less information. However, since the translation of the potential changes into a spike train requires a definition of some arbitrary threshold, we chose to analyze the entire spectral content of the raw signals. Our results therefore represent the effect of the middle ear filtering in comparison to the raw physical echoes on the entire range of spatial frequencies, some of which can probably not be resolved by the bat's auditory system.

The effects of biological filtering are quite salient: it increases the spectral energy at the large scales and attenuates the energy at the small scales in all filter-bank channels. With the cutoff frequency of the leaky integrator used by us (8 kHz) the energy becomes very small (less than 1% of the total energy) for scales smaller than ca. 5 cm. A lower cutoff frequency, as suggested by some authors [17], is expected to make this effect even stronger such that the energy will become very small for even larger scales.

#### Modeling the statistics of plant echoes

We tested different spatial distributions of point reflectors and compared the temporal statistics of the created echoes to those of the real data. We used the Kullback-Leibler (KL) divergence as a measure for goodness of fit. The smaller the KL divergence is, the more similar the distribution of the artificial data is to the observed one.

**Figure 8:**
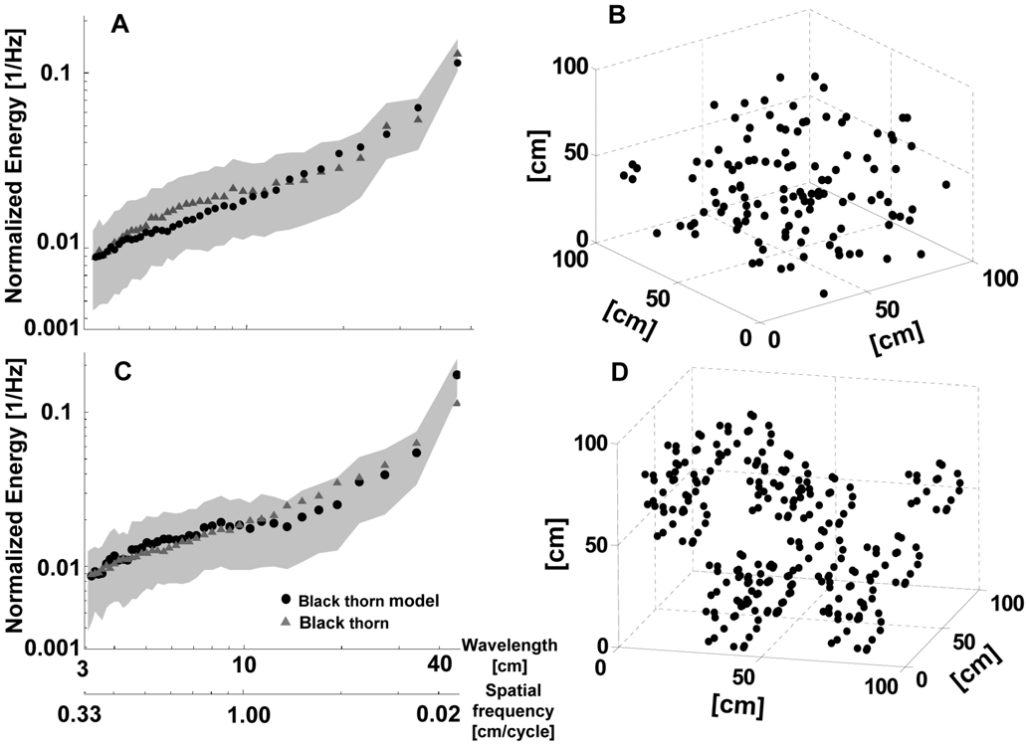
Modeling echo statistics. A) Mean normalized power spectra of blackthorn and the best fitting uniform model for all scales (d_1_ = 16 cm). The shaded area depicts the standard deviation predicted by the model. B) A sample from the uniform 3D Poisson distribution of reflectors with a distance of d = 16 cm. C) Same as A, for the best fitting second-order model (d_1_ = 40 cm, d_2_ = 16 cm, r_2_ = 50 cm). D) A sample from the 3D distribution of reflectors created by the second-order model with the same parameters as in C.

The inverted sigmoid shape that characterizes the natural spectra could be reproduced by the simple uniform model that treats a plant as a 3D array of point reflectors distributed with a characteristic distance between them (Figure 8). In the uniform model, the KL divergence as a function of the distance between reflectors (d_1_) was a concave function with a minimum (representing the best fitting model) at the same distance (20 cm) for all species except apple (Table 1). The second-order model generated sigmoid curves that fit the real data even better than the uniform model for all species except apple as is reflected by lower values of the KL divergence (Table 2). As in the uniform model, the most suitable parameter set (d_1_, d_2_, r_2_) was identical for all species except for apple.

**Table 1:** Uniform Model Optimal Parameters.

Plant Species	Spruce	Blackthorn	Apple	Beech
d_1_ (cm)	20	20	16	20
KL divergence	1.5	1.4	2.0	1.5

The minimum KL values and the corresponding distance parameter (d_1_) for the uniform model when fitting all scales or only large scales.

**Table 2:** Second-order Model Optimal Parameters.

Plant Species	Spruce	Blackthorn	Apple	Beech
d_1_ (cm)	40	40	40	40
d_2_ (cm)	4	4	8	4
r_2_ (cm)	75	75	50	75
KL divergence	0.9	1.2	2.0	1.4

The minimum KL values and the corresponding parameters (d_1_, d_2_, r_2_) for the second-order model when fitting all scales or only large scales.

## Discussion

The spectral echo statistics of the plant species we examined behaved as expected from the cross sections of their leaves (see methods). The spectra of the three broad-leaved species were much more similar to each other than to the single conifer species. Among them, species with larger reflectors (beech and apple) had more energy in the low frequencies (<40 kHz) and less energy in high frequencies (>60 kHz) in comparison to the species with smaller reflectors (blackthorn). Blackthorn echoes had higher energy in the intermediate frequencies (>40 & <60 kHz). Most of the energy reflected at a certain frequency returns from reflectors that are in the optic domain for that frequency (see methods). Apple and beech trees have a larger proportion of reflectors that are in the optic domain in the low frequencies (<40 kHz) while the reflectors of black thorn are mainly in the resonance domain at low frequencies. This results in a higher proportion of energy in the low frequencies in the echoes of apple and beech trees. The higher energy in the intermediate frequencies in black thorn is probably due to the higher proportion of its reflectors in the optic domain at this range of frequencies. The conifer spectra (spruce) contain less energy in the low frequencies and more energy in the high frequencies, compared to the broad-leaved species. For a detailed analysis on how these differences could be used to discriminate between species see Yovel et al [18].

The temporal statistics of complex echoes of different plant species show both robust similarities as well as interpretable differences between them. On a doubly logarithmic plot the averaged power spectra of the IR envelopes of all plant species we examined had an inverted sigmoid curve (Figure 5). The robustness of this pattern is comparable to that found for natural images [5]. Control experiments confirmed that distances between reflectors that are more frequent than others correspond to higher energy at the corresponding wavelength in the spectra of the IR envelope. The fine differences between the sigmoid of different species suggest an interpretable relation between the echo statistics of a complex plant and the spatial arrangement of its reflectors (branches and leaves). Among the three broad-leaved trees for instance, the average spectrum of blackthorn has the least energy in the first domain (10–50 cm) as one would expect from its bush-like shape and lack of large, spread-out branches. Spectral features of an echo convey information on the cross section of a plant's reflectors. Since each plant has a wide range of reflector sizes and since the reflector sizes of different plant species and specimens largely overlap, these features can only provide limited object specific information (e.g., for classifying conifers from broad leaved plants). The ability to use the temporal characteristics of the echo however, could provide additional information that might enable classification of plant species or of specific plants (e.g. for landmark recognition) independently of their size and to a certain extent of their distance.

The spectrum of a single plant echo envelope is much noisier than the average sigmoid curves presented in the results (Figure 3). In order to estimate the minimum number of echoes that have to be averaged to generate the characteristic sigmoid statistics we gradually reduced the numbers of echoes (of a single specimen) and visually tested whether the sigmoid curve was still apparent. We found that as few as 10 echoes of a single plant (acquired from adjacent angles as explained in the methods) are sufficient to generate the sigmoid curve.

When classifying a large object such as a tree while flying past it, a bat could collect several echoes of the object taking into account the time needed to pass the object and an average time interval between emitted calls. Although it has been shown both theoretically [19] and behaviorally [16] that bats can classify complex echoes based on a single echo, it is clear that several echoes might help to emphasize the statistical features that are advantageous for classification. This was already shown in a theoretical approached used by P. Stilz (un-published data) and is supported by our analysis here. In addition the bat might change its calls (see below) in order to improve the acquired information after getting a crude representation using the first calls. This is similar to how the visual system has to saccade in order to focus on different parts of an object that is being classified.

### 

#### The effect of the emitter on the statistics of plant echoes

The analysis of an artificial IR with an equal number of reflectors in all distances suggests that the logarithmic decrease of energy at the small scales (<3 cm) mainly results from the convex energy distribution of our emitted signal. This bias results from the partial ability to deconvolve an echo with a signal of limited bandwidth which acts as a low-pass filter. A larger bandwidth, such as in the ideal bat sweep we simulated, reduces this bias. The differences between the first two domains (50–3 cm) however might still be attributed to the different domains of scale in the plant (e.g, distances between branches vs. distances between leaves). This implies that most of the information that is relevant for the bat in the echo, for instance for classification, is created by large scale structures.

FM Bats will also be subject to a bias of this sort since their down-sweep is never the ideal one. The matched filter that we used for this analysis is optimal to resolve temporally adjacent glints. Thus, even if bats do not perform a direct deconvolution of the echo (i.e., using a coherent or non-coherent matched filter) the signal they receive will be biased towards large scale structures. The extent of the bias will depend on the bandwidth of the calls they emit. Some bats species can manipulate the band-width of their calls by up to several tens of percents. In addition different bat species emit calls with different spectral modulations that might enhance or attenuate the relevant features of plant echoes' temporal statistics (see [20] & [21] for a review of bat call variability). Bats could exploit this range of variability in order to improve classification performance. An additional important parameter in this context is the duration of the call. We did not test the effect of the call duration because the one we used (4 ms) is typical for bats flying at the relevant distance from vegetation and a small change of the duration should not have a major influence on our results.

#### The effects of the receiver on the statistics of plant echoes

The effects of the biological filtering of the auditory system are quite salient: it increases the energy at the large scales and attenuates the energy at the small scales in all filter-bank channels. With the cutoff frequency of the leaky integrator used by us (8 kHz) the energy becomes very small (less than 1% of the total energy) for scales smaller than ca. 5 cm. Interestingly the scale range that is amplified by the auditory system coincides with the range where information for classification should be of the highest relevance for the bat, due to the limited bandwidth of its emitted signal (see above).

The increase of energy in the large scales is visible in all filter-bank channels. Despite this consistent pattern, the different channels emphasize different features of the echo (Figure 7D). Generally speaking, channels with higher characteristic frequency have less energy at large scales and tend to be noisier. These results can be given several physical reasons: On the one hand high frequencies are attenuated more than low ones, thus contributing less to large scale structure and on the other hand low frequencies suffer more scattering, therefore having less influence on the fine temporal structure of the echo. The representation of different features of the echo in different channels, as is done by the auditory system, might simplify classification by emphasizing the characteristics of large-scale structures in the corresponding channels.

#### Modeling the statistics

A very simple model that only considers the spatial arrangement of the plant reflectors was able to reproduce the observed typical sigmoid shape of the IR envelope spectra. This implies that the spatial arrangement of the reflectors is indeed the most important factor in shaping the temporal echo statistics when using a certain emitted call. Several findings suggest that the model was able to capture the basic relation between the structure of the plant and its echo statistics. Increasing the distances between reflectors an adding branch-like clusters improved the total fit of the real data and improved the large scales fit even more (compare KL values in Tables 1 and 2 and see Figure 6).

The optimization of the model parameters clearly revealed the different nature of apple trees in comparison to the other three species (Tables 1 and 2). While apple trees were best modeled by a unique parameter set, spruce, blackthorn and beech were assigned the same parameters although they have a very different spatial structure. This means that even the second-order model is not rich enough to reveal the differences between all four species. Perhaps this is not very surprising since real plants have much more than two orders of scale. Testing higher-order models might lead to different parameters for all species.

#### Use of plant echo statistics by bats

In previous work, we already showed that classification of plant species is possible based on both spectral and temporal features in their echoes [19]. The goal of this work was not to test classification, but to study the statistics of natural plant echoes. Classification is certainly one of the main ways the bats could use the statistics presented here, but not necessarily for species classification. One could argue that classifying a specific individual from other individuals of the same species is sometimes more relevant for a navigating bat (to use as a landmark for instance).

Our previous work found that spectral cues and low frequency temporal cues (energy distribution at certain time points along the echo) are advantageous for a linear classifier of the sort we were using. The findings in this work provide an explanatory basis for those conclusions. Here we clearly showed how the frequency response of a plant and the temporal modulations created by the large scale structures can be related to its physical structure.

### Conclusions

In this work we demonstrated on the one hand the existence of an interpretable relation between the physical world of plants and the statistics of their echoes, and on the other hand found that the general temporal statistics of all plants follow a common pattern. The robustness of this pattern suggests that the sensory system could have evolved in a way that improves the extraction of relevant information.

Previous examinations of the power spectra of natural images [3] revealed a similarly surprising regularity: the spatial frequencies of all natural images, regardless of their content, follow a 1/f^2^ distribution. This finding was then used to further study the statistics of natural image statistics [4] as well as to explain the filtering properties of neurons in the early stages of the visual system [4]–[8]. One of the main hypotheses that was also partially confirmed experimentally about the function of the processing in the early stages of the visual system is that they aim to maximize some measure of information content in the processed images.

Here, we present the first analogous results on the statistics of natural plant echoes. These echoes should be of major importance to more than 800 species of echolocating bats, and might have influenced the evolution of their auditory system. Our results point in a similar direction to the findings for natural images. We find that the information that is enhanced by the bats' active sensor (i.e. the emitted signal) and moreover by the filtering of the early stages of their auditory system coincides with the most reliable domain. Depending on the angle of ensonification, the depth distance between two reflectors as measured from the echo will usually appear to the bat shorter than the actual distance between them. This effect is similar to objects that are far from each other in 3D but appear near in a flattened 2D image. In echolocation, the depth equals the actual distance only if both reflectors are aligned with the ensonification axis. Small scales are thus over-represented in the echoes reflecting both real small scale structures and large scale structures ensonified from a non-perpendicular angle. The small scale information is therefore less reliable when attempting to decipher the structure of the plant. Large scale structures however, represent real large scales and are expected to be less commonly observed in the echoes. In summary, large scale structures are most informative, since they convey information on the differences between plants and also most reliable since they convey real information about the spatial structure (these two features are related).

Both the emitted signal and the auditory system function as low-pass filters in the relevant frequency range, enhancing large scale information. Interestingly, the scale range they enhance corresponds exactly to the one that is most informative as just described. The border between the less and more informative structures (<5 cm) is a result of the model parameters and the emitted signal we used. In contrast to vision, in echolocation, this border can be actively altered by the bat. It can be shifted towards smaller scale structures by increasing the bandwidth of the emitted signal, by shortening the signal or by using higher frequencies and vice versa [22]. It can also be shifted towards smaller scales by shortening the time integration properties of the auditory model. Previous researchers suggested that the large bandwidth is advantageous since it provides a wider range of spectral information revealing the frequency response of an object [15]. Here we show that since a larger bandwidth reduces the low-pass properties of the deconvolution, it would be also beneficial for classifying complex objects.

## Methods

**Figure 9:**
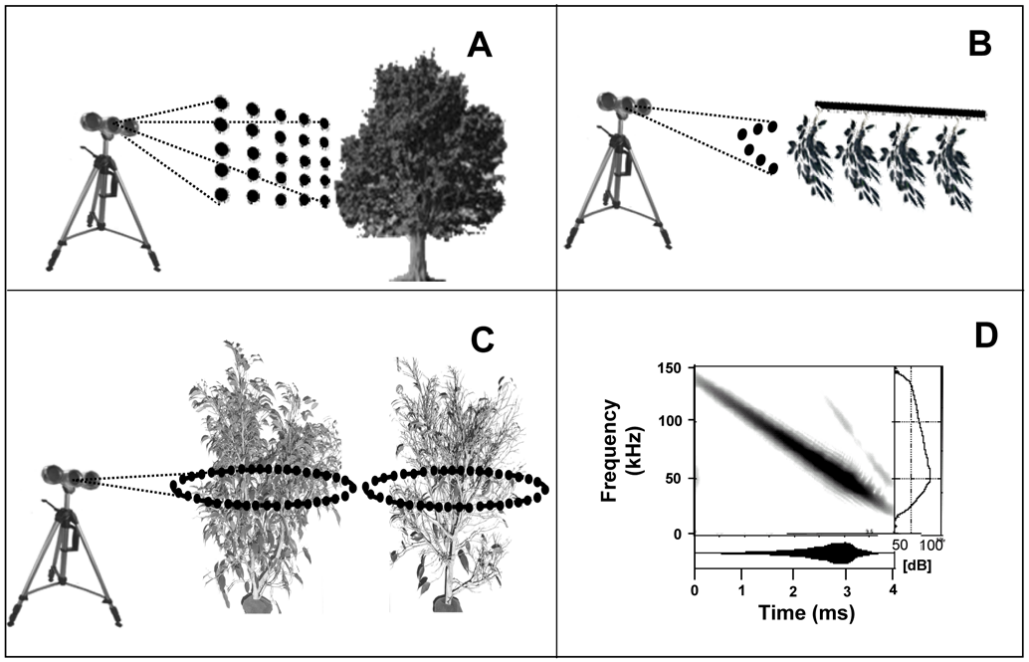
Summary of methods. A) Field data acquisition setting. The grid of points denotes the 25 acquisition positions used for each specimen. B) Ensonification of a plastic model plant from a single elevation angle and 5 horizontal angles. C) Ensonification of a Ficus plant with decreasing leaf density from 36 angles around the plant. Examples of 100% and 5% leaf coverage are shown. C) Time signal and spectrogram of the emitted signal.

All echoes were acquired using a bat-mimicking ultrasonic system consisting of a sonar head with three transducers (Polaroid 600 Series; 4-cm-diam circular aperture) connected to a computer. The sonar head was mounted on a portable tripod. Its central transducer served as an emitter (simulating the bat's mouth) and the two side transducers functioned as receivers (simulating the ears). Backscatter received from the emitted signal was amplified, A/D converted, and recorded by a computer. The emitted signal resembles a typical frequency-modulated bat call in terms of its duration and frequency content (Figure 1A). The emitted signal (Figure 1D) consisted of a four millisecond linear down-sweep from 140 to 25 kHz. We excited the emitter with a constant frequency distribution, but due to the frequency response of the speakers a uni-modal response function was created with a maximum around 50 kHz, providing an intensity of 112 dB (SPL) at the maximal frequency at a distance of 1 m from the emitter. Most of the signal energy was contained in the frequency band between 25–100 kHz. The combined frequency response of our emitter and receivers resulted in a frequency response that resembles a typical frequency-modulated bat call. In contrast to bats, our emitted sound pulse had a rather narrow beam width, with its first null at 50 kHz occurring around 15°, narrower than known for bat calls [23]. The recorded back scatter or echo (both terms will be equally used in this paper, Figure 9B) was digitized at a sampling rate of 1 MHz with 12 bit resolution.

### Field recordings

We recorded the echoes of four plant species, representing common species in the environment of the bats of central Europe. We recorded 50 specimens (always in the field) of each of the following plants:

- Apple tree (*Malus sylvestris*) – This species has large leaves (with an average length of ∼8 cm), in a spacious arrangement. The trees were covered with fruit.
- Norway spruce tree (*Picea abies*) – This was the only conifer tree that was ensonified. Its branches spread homogenously and are evenly covered with needles (very small reflectors in comparison to broad-leaved species). We refer to it as spruce throughout the paper.
- Common beech tree (*Fagus sylvatica*, referred to as beech throughout the paper) – The species is characterized by large flat leaves (with an average length of ∼8 cm) that are usually arranged in the same plane on each branch.
- Blackthorn tree (*Prunus spinosa*) – This species has smaller leaves than the other broad-leaved trees (with an average length of ∼3 cm), without any specific orientation. This species is usually found in a hedge formation rather than as a single tree.

Each specimen was recorded from 25 different aspect angles on an equally spaced 5×5 grid centered at the horizon and the midline of the tree (Figure 9A). This was done by starting at the topmost left point of the grid, 10 degrees above the horizon and 10 degrees left to its midline and then turning the sonar head to the right in sequential steps of 5 degrees along the 5 points of the first row. Next the head was lowered by 5 degrees and the procedure was repeated, this time towards the left. This procedure provided us with 1250 echoes for each species from each ear. The distance between plant and tripod was always 1.5 m, and the height of the tripod above ground was set to 1.35 m. The same echo database was also used in our previous study on ultrasonic plant classification [18].

### Indoor experiments

To understand the relationship between plant structure and corresponding echoes we conducted two indoor experiments:

- As a very simple test of our assumptions we ensonified a plastic model plant that was composed of four identically leaved twigs mounted on a main stem (∼1.5 m long, Figure 9B). We repeated this experiment, changing only the distance between the twigs. Each branch (defined by its specific twig distance) was ensonified from a single elevation angle, ca. 10 centimeters higher than the branch and almost perpendicular to the surface of the twigs, and from six approximately equally spaced azimuth angles covering a total sector of ∼45 degrees around the branch.
- In the second experiment we ensonified a single potted plant of the species *Ficus benjamina* while randomly ripping off its leaves, i.e. decreasing its leaf density (Figure 9C). The leaf density was decreased in 11 steps starting from 100% leaf coverage going down to ∼0%. At each step, the plant (∼2.5 m high, ∼1.5 diameter, initially with ∼1200 leaves) was ensonified from the horizontal plane (parallel to the floor) and from 36 approximately equally spaced azimuth directions surrounding it.

### Spectral analysis

All signal processing was performed with Matlab 7.0. To analyze the spectra of the echoes their power spectral densities (PSD) were computed using Welch's method with a 5 ms window and an overlap of 50%. The PSDs (referred to as spectra) were normalized to a sum of one so that the value for each frequency corresponds to the proportion of energy in this frequency. Since the spectrum of the emitted signal was always identical, differences between the spectra of different species imply structural differences and particularly differences in the cross section of the reflectors [see [17] for a general review and [18] for a specific discussion of these species).

### Temporal analysis

In order to investigate the relationship between the spatial arrangement of the plant reflectors and the corresponding echo, all echoes went through the following analysis steps:

1. Cross-correlation with the emitted signal to approximate the IR of the plant.
2. A Hilbert transform for calculating the envelope of the IR.
3. The PSD of the envelope using Welch's method with an 8 ms window for the long outdoor echoes and a 4 ms window for the indoor echoes and a 50% overlap for both. The PSDs were observed only down to a scale of ∼1.5 cm since, as is demonstrated in the discussion smaller scales are highly influenced by artifacts. The spectra were normalized to a sum of one as in the PSDs of the plant echoes. This analysis method enables an observation of the echo-structure relation independently of the cross section of the reflectors. Notice that while for the spectral analysis we calculated the PSDs of the original echoes, for the temporal analysis we did so for the envelope of the IR.

### Modeling temporal statistics

#### The uniform model

The uniform model creates a three-dimensional Poisson distribution of point reflectors (simulations were done in Matlab 7.0). It has two parameters: 1) d_1_ - the typical distance between the reflectors which can be easily translated into the λ parameter of the Poisson distribution, depicting the probability to find a reflector in a cubic volume with a side length of 0.5 cm (the basic unit of volume we used). 2) L – the side length of the entire cube ensonified by the signal. The first parameter was fit (see below) while the second was estimated from the time duration of the original echoes.

#### The clustered models

These models include more complex non-stationary distributions in which point reflectors are clustered in Poisson clouds. Due to the high complexity of the calculation of these models we only tested second-order models in which spherical Poisson clusters of reflectors were distributed around the centers found according to the uniform model. Higher-order models can be derived in a sequential fashion based on lower-order models by distributing spherical Poisson clusters of reflector around the reflectors of the lower level. Each order of the n-order models adds two parameters to the two initial ones of the uniform model: r_i_ - the radius of the spheres of the i'th order and d_i_- defining the typical distance between the reflectors in each cloud at level i (for the clusters i>1).

#### Optimization of model parameters

We tested models with different sets of parameters (d, r) spread along the entire range of physical plausible values (see below). 500 echoes were simulated for each set of parameters. The spectra of the echoes were computed in the same way as for the real echoes. We used the Kullback-Leibler (KL) divergence to choose the model that best fits the observed real data. Assuming that the different frequencies (scales) are independent we used the KL divergence to compare the probability distribution functions (PDF) of the model and the real data at each frequency. The PDFs of the frequencies of single spectra were highly non-gaussian in all frequencies (both for real data and models) and were therefore estimated by a histogram with 8 equally spaced bins. The similarity between model and measurement was then calculated according to:
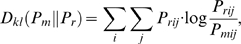
(4)where 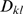 is the sum of the KL divergence for the tested scale range (i). 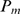 is the PDF of the model spectra and 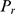 is the PDF of the measurement spectra. The *i*th and *j*th index of both 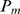 and 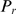 represents the *j*th histogram bin of the *i*th frequency. The KL divergence measures the expected difference in the number of bits required to code samples from 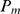 when using a code based on 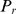. This is known as the relative entropy between the two PDFs and is an estimate of the difference between the two distributions. According to Gibbs' inequality, the KL divergence always satisfies
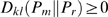
(5)where equality occurs only if 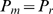. The model with the lowest 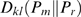 value was therefore chosen as the one that best fits the real data. We tested the fit of the models to the data in the scales 45>i>3 cm since the smaller scales already correspond to the falling tail of the sigmoid (Figure 3) and are probably less informative as described in the discussion. We used the following values for the different parameters: For the uniform model we tested the following parameters: d_1_ = [4, 8, 12, 16, 20, 30]. For the second-order model we tested the combinations of d_1_ and d_2_ as specified in Table 3.

**Table 3:** Second-order Model Tested Parameters.

d_1_ [cm]	60	60	60	60	40	40	40	20	20	10
d_2_[cm]	30	16	8	4	16	8	4	8	4	4

Combination of distance parameters (d_1_, d_2_) used for the second-order model. Each distance combination was tested with the following cluster radii (r_2_): 25, 50 or 75 cm.

#### Auditory system model

To compare our results to the statistics of the echo after going through the biological filtering we applied the standard auditory system model (see [24] for the full details). Basically this model first applies a 60-channel constant Q gamma-tone filter-bank on the echo. Next the response of each of the filter-bank channels is half-wave rectified and subsequently compressed to mimic the first transformations occurring in the inner hair cell. Finally a leaky integrator is used with a fourth-order low pass filter with a cutoff frequency of 8 kHz removing all phase information in the ultrasonic range. We then calculated the spectra of the outputs of this process in the same way as we did for the envelopes of the echo IRs. Note that this provided us with 30 spectra for each echo (one for each channel in the frequency range of the emitted signal).
